# Retraction: Anti-Wrinkle Effect of Magnesium Lithospermate B from *Salvia miltiorrhiza* BUNGE: Inhibition of MMPs *via* NF-kB Signaling

**DOI:** 10.1371/journal.pone.0216473

**Published:** 2019-04-30

**Authors:** The PLOS ONE Editors

Following publication of this article [1], the following concerns were noted:

- Fig 7A ERK panel lanes 3–4 appear similar to Fig 7B ERK panel lanes 1–2
- Fig 7A JNK, Fig 7A p-p38 and Fig 7B p-JNK panels appear to contain horizontal discontinuities above and below the bands
- Fig 5A cJun panel appears similar to the Fig 6A iNOS panel
- Fig 8B bright field and fluorescence micrographs do not appear to show the same fields in panels for Vehicle and MLB 10μM
- Fig 8B: there are similarities between Vehicle and Trolox 10μM bright field micrographs, and between Con and MLB 10μM bright field micrographs

The authors have provided replacement images for all panels in Fig 8 and noted that errors were made in preparing the original figure. The authors note that the background discontinuities in Fig 7 are observed owing to the use of polyclonal antibodies that have lower purity and that detect nonspecific bands. The authors do not agree with the similarities noted in the remaining figure panels and have explained that the primary data underlying all original figures in the article, except those provided for Fig 8, are unavailable.

In light of the unresolved concerns about Figs 5, 6 and 7, the *PLOS ONE* Editors retract this article.

YRJ, DHK, SRK, HJA, EKL, TT, NDK, TY, JNP, HYC did not agree with retraction.
